# Medical Notes and Reflections

**Published:** 1857-05

**Authors:** 


ART. III.—*Medical Notes and Reflections.* By SIR HENRY HOLLAND, Bart., M.D., F.R.S., etc. From the third London Edition, Philadelphia, 1857, 1 vol. 8vo, pp. 493.

IN this smoothly and carefully written volume is recorded, in a perspicuous manner, the professional experience of a thoughtful and observant physician, who has pursued the healing art in the great metropolis of England, for the long period of thirty-six years, during the whole of which time, he has "been accustomed to preserve notes, not merely of particular cases, but also of such general reflections as were suggested to him by actual observation."

In the preface to the first edition of this work, which appeared in 1839, and was republished in Philadelphia, in the same year, the author sets forth the general scope and object of his "Notes and Reflections," in the following paragraph,

"What I would fain hope may be found in this volume is, a just view of some of the relations of diseases, as well to each other as to the healthy functions of the body;—the correction of some doubtful or erroneous views in practice;—suggestions which may be useful as regards particular classes of remedies;—and reflections on certain points of physiology, in which without any pretension to experimental inquiry, it appeared to me that something might be gained by arranging the facts and inferences in a new form. On topics of the latter class I have sought especially to associate pathology with physiology, the morbid with the natural and healthy states of the body; believing this principle of modern inquiry to be, above all others, fertile in sound conclusions, and far from being yet worked out to its full extent. If I might venture on giving any distinctive character to the volume, it would be that of aiming throughout at this object."

Concerning the attempt here referred to, of connecting physiology and pathology, or rather treating these two branches or departments of scientific inquiry, as the inseparable and mutually elucidating parts of the great science of Biology, we have already spoken* in our commentary upon the excellent treatise of Chambers on *Digestion and its Derangements.*

In the present edition of his work, Dr. Holland has attempted to obviate the somewhat desultory character of former editions, by "bringing the chapters more into series, as regards the relation of their subjects." The early chapters treat of certain pathological principles, through which the author has sought to associate together various morbid conditions, not usually thus connected in our systems of nosology. The concluding chapters are mainly occupied with the treatment of disease, and the action of certain medicinal and remedial agents.

The volume very appropriately opens with a chapter on Medical Evidence, the importance of which is only equalled by the difficulty of estimating it aright. Upon the correct appreciation of medical evidence depends medical truth, and the records of the age which is most solicitous of medical truth, must necessarily constitute the brightest chapter in the history of the healing art. Founded upon observation and experiment, wholesomely guided by reason, medical truth is strictly inductive. The complexity, magnitude, and delicacy of the phenomena which it represents, must ever deprive it, perhaps, of a purely demonstrative character. It deals with facts as the mathematician deals with figures, and recognizes no speculation, except those generalizations which incontestably flow from its legitimate records. Mind and matter wedded upon the altar of the organic cell, mind and matter acting and reacting upon each other throughout the incessant processes of development, growth, reproduction, and decay; these are the subjects which the physician is daily and hourly called upon to observe and record. How careful should he be, therefore, that the conflicting evidence thus set before him, do not mislead him, and consequently endanger those who depend upon his judgment for the preservation of their health, or the restoration of that health when lost. If the physician estimates this evidence aright, then he dignifies his profession, and through it, becomes a benefactor to all mankind. Let him through prejudice or carelessness, or inherent inability to grapple with the great problems of his art, fail in reading this evidence correctly, and the mischief daily inflicted will expand like the surface-waves produced by a falling stone, until far-distant points are affected by its malign influences.

Peculiarities of mind and body, the varying aspects which the same disease presents in different individuals, the intimate manner in which primary and secondary causes of disease are blended together, the difficulty of discriminating between the physical, chemical, and vital operations of

* See Med. Examiner, Nov. 1856.

the human organism, the constantly varying effects of remedial agents employed in ameliorating disease, and the consequent difficulty of assigning them their true value,—such are a few of the associated elements which give to medicine its character of complexity and uncertainty—a character so disheartening to the conscientious physician, and so encouraging to the empiricist and the impostor, who avail themselves of this confusion to build upon the many illy-understood and "false facts" of medicine, their systems of fraud and deception, whose chief excellence is to put money in their purse. Only by the right use of medical evidence can we hope to deprive medicine of its uncertainty. Very properly, therefore, does our author introduce it at the threshold of his "Notes and Reflections," calling attention to the light manner in which this testimony is too generally weighed by physicians themselves, the necessity of testing the action and value of a new remedy by a logically severe and cautious method of observation and experiment, and avoiding the *post hoc, propter hoc* reasoning which has so often disgraced the annals of medical philosophy.

Among the sources of error and embarrassment to which all medical proof is exposed, Dr. Holland instances the uncertain notions entertained as to the functions of many important parts of the body, the conflicting views upon contagious diseases, and the uncertainty as to the action of drugs upon the animal economy. He also comments upon the advantages of the numerical method so skilfully employed by Louis, and calls attention to the manifest tendency of the age to busy itself with the abstruse questions relating to vitality, the vis nervosa, the relations of mental and material power, and the obstacles which retard the progress of medical science, in the shape of extreme credulity, on the one hand, and extreme scepticism, on the other.

Hereditary temperament and tendency to disease constitute the subject-matter of Chapter II.

A moment's reflection will show that the laws of hereditary disease are, in great measure, modifications of the laws of specific forms, so laboriously developed by Cuvier, in his philosophical attempt to restore the forms of the fossil world; by Geoffroy Saint-Hilaire, Lamarck, Blainville, De Maillet, and Serres, in France; and by Meckel, Tiedemann, Van Baer, Spix, Oken, Valentin, and others, in Germany. Considered from such a stand-point, the whole subject acquires a highly philosophical character.

"For in considering this hereditary tendency to disease, whether arising from structural or less obvious cause, it is needful to regard it in connection with, or even as part and effect of, that great general principle, through which varieties of species have been spread over the globe, with obvious marks of wise and beneficent design. That there are certain determinate laws, by which the permanence of species is secured, must be admitted as a general fact—the result of all observations hitherto made on organic forms, both living and extinct. The very circumstance of the frequent repetition of analogous and parallel series of forms, under different physical conditions, and at different geological epochs, though admitting some doubt as to its interpretation, is nevertheless most favorable to this opinion. Still we are not entitled to deny to the followers of Geoffroy St. Hilaire the *possibility* that it may be otherwise; and that time may hereafter disclose to us some evidence that species are not immutable. It is, in truth, one of the great remaining problems of natural science, to determine whether the variations induced upon any *specific form* of animal life, can ever become such in amount and fixedness as to constitute a new species, in the complete definition of the term;—a question not to be settled 'within the hour-glass of one man's life,' but requiring ages of continuous observation, even for the chance of solution."

Expressing his belief in the permanence of species,—a belief so boldly, and we may say so successfully attacked by Gérard, in the *Dictionnaire Universelle* of D'Orbigny—and dismissing the notion of a single primitive germ or absolute unity of animal type as a thing not proved, while he admits that comparative anatomy is ever carrying us back, by successive steps, to simpler and more fundamental types of animal life—Dr. Holland thus proceeds:

"It is still a subject of dispute whether this progressive approach to more general forms is stopped by essential differences of type, or whether such divisions do at any point in the series really exist—whether there are separate circles of analogous structure, as defined by the four great classes of Cuvier, or by the more complex systems of some later zoologists—or whether these are but artificial limits, subordinate to a common type pervading all the forms of animal life. These are questions strictly within the scope of research, and the solution of which connects itself with some of the highest objects of natural science. I have never, as already stated, seen cause to think the arguments of Natural Theology, at all affected by these inquiries; even when pushed to the speculations of a primitive germ, or original unity of type. Its truth, indeed, is far above the reach of what, after all, are but subordinate researches, even if they could by possibility attain the proofs of what these terms express. The chain is lengthened, and its parts are connected together by new and unexpected links. But still it is a chain of designed organization throughout; and if we simplify the first of these links, it is but to render more wonderful the number and perfection of the varieties which are evolved in definite forms from this elementary structure. If the parts in man have all their analogies or models in lower animals; or if, according to one view proposed, we regard the several organs of the human embryo as passing through all the gradations of lower types, before reaching their perfect development in man; still the great argument remains the same. The progressive elaboration throughout all its stages is definite and designed;—the points are fixed at which each deviation has its beginning from the more common antecedent forms;—the limit of change, as defined by species, is fixed in each particular case. If there be an argument for the unity of creation, more complete and comprehensive than another, it is that which is furnished by the recent progress of comparative anatomy; enabling the observer, by the uniformity of general laws, to predicate from a single minute part of structure all the more important generic characters of the animal to which it belongs.

"If now, recurring to the subject before us, we make the progress of development the basis of our views, and follow this from more general forms to those which are successively subordinate—even as far as to families and individuals of the same species—we thereby connect the subject of hereditary aberrations with this more general law; finding reason from analogy to expect that such aberrations should occur, and that they should be carried into extreme variety and minuteness. The different parts of structure and function become more special, or individual at each step of descent; never losing, however, their relation to the original common type; and in the instances at least of individuals of the same species, tending ever to recur to it, when the causes of change are modified or withdrawn."

"If peculiarities of external form and feature, whencesoever originally derived, tend so speedily to become hereditary—affecting, as we see on every side, not families alone, but, by intermixture and descent, whole races of mankind—we can have no doubt that deviations of internal structure (whether they be of deficiency or excess, or of any other nature) are similarly transmitted; and with them propensities to, or conditions of, morbid action in the parts thus organized. Though the direct proof is not equal for the two cases, and though the effects resulting are of such different importance, yet it is certain that the peculiarities, so carried on from one generation to another, have reference for both to one common law. Those deviations from the primitive or common type of the species, which occur chiefly in the bony structure, integuments, or muscular fabric, producing varieties in the outward form and feature, in the texture or color of the skin, hair, &c., may exist to great extent, without affecting in any important way the health or natural functions of the individual. On the other hand, much smaller deviations from this type, in the internal organs of circulation, respiration, digestion, absorption, or secretion—or in the brain and nervous system—may produce morbid actions, painful in progress and fatal in result; each class of deviations alike transmissible to progeny, under the same general law. A faulty texture of skin, a hare-lip, or strabismus—all frequent hereditary defects—may be of little import to health; while a morbid structure of internal membranes, a slight organic deviation in the heart, or a tendency to deposit in the crystalline lens, may inflict danger or distress upon every part of life.

"This view, if authentic, is obviously one of singular importance in the history of disease, and capable of very wide application. It throws light upon the connection of various morbid states, by giving the relation to a common physical principle in their cause; and this principle one which is associated with others of the more general laws of life. Here, for example, we must look for explanation of the difference in the average duration of life in different families—a fact well attested in itself, notwithstanding the many exceptions which the laws or social usages of mankind are ever inducing upon it. There is no just reason to doubt that hereditary peculiarities of structure are as frequent and varied—perhaps as extensive—in the internal organs, as in the external parts of the body. Analogy would suggest this as a probable truth. It is confirmed in great measure by observation; and more extensively as the examination is rendered more minute."

The method here advocated, of pursuing the study of hereditary disease through the unity or analogy of structure in corresponding organs, is undoubtedly the natural one, and therefore most likely to lead to correct conclusions.

After inquiring whether bodily peculiarities, disease, &c., are transmitted by the blood or by the solids, the author proceeds to lay before his readers a number of singular and highly interesting cases of hereditary malconformation and tendency to disease, concluding the chapter with some speculations upon atavism, and the tendency manifested by many diseases to evolve themselves at particular periods of life.

Passing over Chapter III, in which is indicated the method of inquiry as to contagion,—a method which he treats under the three heads:—1st. The state of the person giving the infection; 2d. The state of the person receiving it; and 3d. The condition of the medium through which the transference is made,—we come to the subject of "Diseases occurring but once in life," which is discussed in Chapter IV with the following conclusions:

"*First*, That the series of actions or changes, constituting these diseases, have their seat in the blood, and are carried on throughout by the circulation.

"*Secondly*, That, although we have no cognizance of the actual physical state or changes which give protection against their occurrence or recurrence, there is more reason for seeking these in an altered state of the blood than in any other part of the animal organization.

"*Thirdly*, That the conditions which render one disease incompatible with another—or produce the various results of their mutual interference—or limit to man their production and development—all probably depend on the same physical principles as the simpler fact of immunity, and have equally their source and seat in the blood.

"*Fourthly*, That the state of immunity, on whatever bodily conditions it depends, is liable to fluctuation in various ways;—is often partial and imperfect when first obtained—occasionally diminished or lost by time, even where there is presumption of its having been most complete at first—possibly affected in some degree, and after a certain lapse of time, in all;—the rate of such changes being different in different persons, and modified also by the access of other diseases, or by the contingencies of life.

"*Fifthly*, That a constitutional or hereditary tendency exists throughout all these phenomena;—modifying susceptibility—determining, in conjunction with other causes, the regular or irregular course of the disease—affecting its degree of virulence—and giving liability to its recurrence; and that this habit is not limited to families, but extends even to larger communities.

"*Sixthly*, That the several modifications, above named, depend further on the quality or quantity of the virus received in each case—conditions admitting of being infinitely varied; and giving, in conjunction with the effects of season, locality, and human constitution, the peculiar character which every epidemic more or less assumes."

Chapter V is devoted to the consideration of the connection and classification of measles, scarlatina, hooping cough, infantile remittent fever, and to the epidemic constitution of seasons.

"I have observed repeatedly that, during the seasons in which influenza has occurred as a general and severe epidemic, these two diseases of scarlatina and measles have been more than usually frequent; though in no instance, perhaps, reaching the extent to which they occasionally occur at other times;—and further, that during the actual prevalence of influenza, a class of cases has appeared of very singular and ambiguous kind, having many appearances analogous to each of these disorders, but particularly, as I think, to scarlatina. I noticed frequent cases of this nature during the influenza of the spring of 1833, when the scarlet fever was also very prevalent, and produced much mortality in London. A peculiar spotted efflorescence on the skin, generally attended with some angina, and occasionally with slight ulceration of the throat, were the symptoms principally marking this apparent relation; which was sufficient in degree to suggest the question, whether it arose from the concurrent action of two morbid causes; or from one virus capable of producing different forms of disease, according to the texture on which it fell, or other less obvious circumstances. If hazarding an opinion on this obscure subject, it would be in favor of the former view. Though we have many instances of such seeming incongruity of two diseases, that one present in the system precludes the ingress of the other, still there is no foundation for a general law to this effect. On the contrary, we have various proof that morbid actions, derived from sources wholly different, may coexist in the body; severally modifying each other, though under conditions scarcely even surmised in our present knowledge.

"Erysipelas and erythematous inflammations are frequently found to prevail during particular seasons, and in certain localities; and with many common characters. Such are the affection of internal membranes, the translation of the inflammation to the skin, the frequent presence of fever of low or typhoid type, and the influence of this state of habit upon all casual injuries or disorders of the body occurring at the time. Instances of this kind were formerly more explicitly recorded, as the writings of Sydenham testify, in connection with the doctrine of an *epidemic constitution* of seasons—a theory which scarcely receives its due regard in our own day.

"A further example may be found in a sort of epidemic character which puerperal fever often takes, in seasons when erysipelas is very prevalent; rendering it probable that there are some causes in common to the local and constitutional disease;—if not indeed the same morbid cause to both, modified in its action by the texture affected, and by the individual peculiarities of each case. The presumption, now rendered almost certain, that both puerperal fever, and erysipelas in its ordinary character, become contagious under certain circumstances of type, season, or locality (and these seemingly alike in different instances), may be admitted in further proof of such a relation. And further, we have reason to believe that there is increased tendency to both disorders during the prevalence of epidemic influenza; as was well attested with regard to the puerperal fever in the spring of 1838.

"The hooping-cough among children has also been singularly concurrent with some of the epidemics already mentioned; particularly, as I find in my notes, with the influenza, which spread so universally over England in the summer of 1831, immediately before the first appearance of the Asiatic cholera in this country; and again very remarkably with the similar, though less severe epidemic, which prevailed in London during the spring of 1838. The cases were numerous at both these periods, in which, in children especially, it was scarcely possible to draw distinction between the two disorders; and difficult to avoid the persuasion that some cause was concerned, analogous or common to both."

The prevalence of infantile remittent fever during influenzal epidemics; the connection of epidemic pertussis with an unusual tendency to bowel affections; the singular frequency, especially among children, of ulcerations or eruptions of the mouth, fauces, nose, lips, and face, during the prevalence of these bowel disorders; the relations of dysentery to infantile fever; the connection between epidemic dysenteries and certain pestilential affections, such as typhus and typhoid fevers, are successively discussed in a detailed and instructive manner. This chapter terminates with a short commentary on the various and conflicting systems of nosology, which at various periods in the history of medicine, have had their strenuous advocates. In the science of physiology, the author finds the true basis for the classification of disease.

"Classification may be sought for, either in the particular character of the morbid actions which produce disease, or in the organs and functions themselves, viewing them as severally subjected to these morbid actions."

"Of the two main principles just stated as the basis of classification, that resting upon functions may, I think, be reasonably preferred. The nature of morbid actions, however, much as our views have been enlarged, is still in numerous points ambiguous and obscure. Until the true theories of fever and inflammation are more entirely established, and the diseased actions of the nervous system more exactly defined, we bring incertitude into all arrangements of which they principally form the basis. It is true that these difficulties appertain to every system; the conditions out of which they arise, being in one degree or other common to all. But being such as they are, we cannot look to find here any certain basis for the classification of disease, though much that is valuable in approximation to it.

"A system of Nosology, of which function as connected with organs is the basis, has various advantages in practice. Less ambiguous in its foundation, it connects more explicitly the healthy and morbid states of the organs; admits more readily all the changes which the progress of knowledge may require; and often itself suggests the methods and direction of this progress. We must admit that the several functions of life are not all equally or exactly defined. In disease, moreover, it rarely happens that one function only is concerned; or if such be the case in the outset, others for the most part get involved either by metastasis or extension of morbid action. Still there are few systems of classification, whatever the subject, which do not labor under similar difficulties as to the bases on which they rest. Those may be deemed in every case the soundest in principle which contain within themselves the elements of rectification, and best facilitate, meanwhile, the advancement of science."

The theme of Chapter VI, "Disturbed Balance of Circulation and Metastasis of Disease," is wholly physiological in its character.

In Chapter VII, the longest and perhaps the most interesting in the whole work, the author treats his readers to an essay on the "Influence of Weather in relation to Disease."

When we reflect that the elements of climate constitute the external conditions of vitality, and that these elements are numerous and within certain limits constantly varying in intensity and in combination, and consequently in the influence which they exert upon the manifestations of life in both the animal and vegetable worlds; when we reflect that they are associated in such a manner as to render it extremely difficult to determine their respective, individual actions; and when, moreover, we call to mind that the living organism upon which these actions are exerted, is also made up of a series of elements, the true character and value of each of which are so difficult to determine, we at once become aware of the complexity as well as of the magnitude of the subject, and the important part which climatic science must ultimately play in solving many of the obscure and perplexing questions of pathology. Indeed, a progressive physical science in the careful hands of a few illustrious men, is already casting a clear and steady light upon the hitherto dubious paths of the physiologist and pathologist; and these latter, after years of prejudice and apathy, are at length beginning to perceive in the principles and exact logical methods of the former, the true key to the complex problems which constitute their daily studies.

Among the many interesting topics alluded to in this chapter, we may briefly mention, for example, the direct effects of temperature upon the body; the degree to which external cold may alter the balance of the circulation,—directly, by contracting the capillaries and smaller arteries of the surface, or indirectly through the effect of this altered equilibrium upon the action of the heart itself; the tendency of sudden changes of temperature to produce topical inflammation, through irregularities of the circulation, resulting from general or partial changes in the superficial capillaries; the influence of external temperature on the functions of the skin, whether those of transudation or simple evaporation; the effects of a sudden arrestation of the perspiratory function by external cold; the direct action of cold as a remedy; the relation of atmospheric temperature and electricity to endemic and epidemic diseases, &c. &c.

Chapter VIII is appropriated to the consideration of dietetics, and the disorders of digestion.

The subject of gout as a constitutional disease claims the attention of the reader in Chapter IX. According to Dr. Holland, the greater portion of our information of the intimate nature of this disease, may be comprised under the following general heads :

"1. That there is some part of bodily organization disposing to Gout, because it is an hereditary disorder.

"2. That there is a *materies morbi*, whatever its nature, capable of accumulation in the system,—of change of place within the body,—and of removal from it.

"3. That though identity be not hitherto proved, there is a presumable relation between the lithic acid, or its compounds, and the matter of Gout; and a connection through this with other forms of the calculous diathesis.

"4. That the accumulation of this matter of the disease may be presumed to be in the blood ; and its retrocession or change of place, when occurring, to be effected through the same medium.

"5. That an attack of Gout, commonly so called, consists in, or tends to produce, the removal of this matter from the circulation ; either by deposits in the parts affected ; by the excretions ; or in some other less obvious way, through the train of actions forming the paroxysm of the disorder.

"6. That there is intimate relation between the condition of gouty habit, and the functions of the kidneys and liver, both in health and disease.

"7. And that the same state of habit, or predisposition, which in some persons produces the acute attack of Gout, or slower deposits about the joints, does in others, and particularly in females, testify itself solely by disorder of internal parts, including the nervous system, as well as various secreting and excreting organs of the body."

But the limits which are assigned to this notice, being nearly filled, we must hasten to conclude by stating that a chapter is appropriated to each of the following topics :—Morbid Actions of an Intermittent kind, The Medical Treatment of Old Age, Recent Epidemic Influenzas, Prognosis as a part of Practice, Pain as a Symptom of Disease, Points where a Patient may Judge for Himself, Methods of Prescription, Internal Hemorrhages and Morbid Secretions as the Subjects of Medical Treatment, Some supposed Diseases of the Spine, Hypochondriasis, The Exercise of Respiration, Some Points in the Pathology of the Colon, Abuse of Purgative Medicines, Bleeding in Affections of the Brain, Use of Emetics, Uses of Diluents, Sudorific Medicines, Use of Opiates, Mercurial Medicines, Use of

Digitalis, Antimonial Medicines, Hypothesis of Animalcule Life as a Cause of Disease, and Cholera.

Upon the whole, the work of Dr. Holland may be regarded as an agreeable and useful addition to the library of the physician. More suggestive than original, its true merit lies in the elevated character of the various topics embraced in its pages, and the philosophical tone in which they are discussed. To the student just entering the profession, and to those yet young in its practice, the volume under consideration recommends itself, not only for its general avoidance of those technicalities which so often prove incumbrances, rather than auxiliaries to the progress of medical science; but also for the careful manner in which the author attempts to define the limits of our information concerning the various topics treated of, by "separating what is actual and assured knowledge, from that which but usurps the name and show of it."

A prominent feature which meets us here and there, throughout the work, is the great stress which Dr. H. lays upon the relation of medicine to the physical sciences in general. In his concluding remarks, this connection finds expression in the following language, with which this brief notice—written *currente calamo*—must terminate.

"I cannot, however, close what I am now writing, without some reference to another object, of which I have never lost sight in the consideration of these subjects. I mean, the relation of Medicine, both as a science and system of practice, to the other physical sciences—a connection ever tending to closer approximation; and through which its progress to higher attainments and powers is mainly to be looked for in the future. This is a field for exertion so vast and various, that every physician may fulfil some part in the required labor, by observation, experiment, or reasoning. Chemistry in its every branch (but especially the Chemistry of Organic Life, associated, as it now is, with the doctrine of definite atomic proportions) stands foremost in the connection thus established. But Electricity, Optics, Meteorology, Natural History, and even the mechanical sciences, severally contribute towards this great circle of relations; which, while unceasingly enlarged by new discoveries, is at the same time ever concentrating itself around common laws and higher generalizations. To these I have often had occasion to refer in the preceding chapters; and have indicated, wherever able to do so, the means by which their association with medical knowledge may best be enlarged and defined.

"For from the growth of the other physical sciences, Medicine has not only enlarged its own sphere, and gained a sounder philosophy, but it has also acquired new methods and instruments with which to conduct future inquiry. Numerous and exact records now give effect to that happy method of averages, which we have noted as being an almost mathematical guide to truth. Every well-attested case thus acquires its appropriate place and value; and facts, before lost or dispersed, are now virtually registered in the conclusions to which they have contributed. Of the direct instruments which science has furnished in aid of medicine and physiology, the Microscope is the most consummate and wonderful example. But other refined applications of physical agents have become familiar to our use; giving the power of looking more deeply into the interior of the living frame; of better determining its morbid conditions; and of applying remedies more closely and effectually for their relief.

"To remove error—to perfect what is incomplete in knowledge—and to attain by discovery what is new—may fitly be placed before the young medical man, as objects not merely compatible with the practical duties of his profession, but as even aiding their more entire fulfilment. Few indeed possess the means requisite to command fame or success in such pursuits. But the objects themselves need not, and ought not, to be alien to any condition of medical life; and the endeavor to attain them, to whatever extent it may in each case be possible, will ever form the best guarantee for the just and honorable discharge of professional duty."

